# AGING AND THE MICROBIOME

**DOI:** 10.1093/geroni/igae098.1591

**Published:** 2024-12-31

**Authors:** Shuo Han

**Affiliations:** Chair

## Abstract

This 90-minute session will explore key interactions between aging and the microbiome leveraging studies in animal models and humans. The three invited talks will discuss the following diverse topics in microbe-aging interactions: 1) functional roles of gut bacteria and small molecules in host aging and physiology in C. elegans and mice (Han); 2) gut microbial and metabolic changes across lifespan and how these changes impact brain, cardiovascular, and offspring health (McCullough); and 3) gut microbiome contributions to age-associated cognitive decline, inflammation, and immune functions in older adults, based on the outcomes from the Microbiome in Aging Gut and Brain consortium study (Yadav).

